# Development and feasibility testing of a play-based psychosocial intervention for reduced patient stress in a pediatric care setting: experiences from Pakistan

**DOI:** 10.1186/s40814-021-00781-8

**Published:** 2021-03-06

**Authors:** Muneera A. Rasheed, Vardah Bharuchi, Waliyah Mughis, Ayesha Hussain

**Affiliations:** 1 Centre for International Health, Department of Global Public Health and Primary Care, University of Bergen, Bergen, Norway; 2 Department of Paediatrics & Child Health, Aga Khan University, Karachi, Pakistan; 3 Teach for Pakistan, Karachi, Pakistan

**Keywords:** Hospitalization, Intervention development, Nurturing Care Framework, Play therapy, Psychology trainees

## Abstract

**Background:**

Hospitalization can be a source of great stress for children and their families. In high-income countries, there are specialized staff to help children cope using different techniques including play. However, it is a major challenge in low and middle-income countries (LMIC) due to financial constraints and untrained human resource. The objective of study was to develop and test the feasibility and acceptance of a psychology trainee-delivered model postulated on employing play as a means of enhancing child-parent interactions leading to reduced stress during hospitalization.

**Methods:**

This study was conducted in the paediatric ward of a tertiary care private hospital in Pakistan. Pre-intervention survey revealed that parental stress stemmed mainly from seeing their child irritable, distressed, or in pain. Using a theory of change model, a play-based psychosocial intervention was developed to address these factors. The intervention approach was informed by principles of Nurturing Care Framework and play therapy. Children between birth and 6 years admitted in the acute care ward were eligible. The intervention was delivered between March 2019 and December 2020 by psychology trainees who were supervised by a clinical psychologist. The play-based sessions were delivered at the bedside and ranged from 20 to 40 min. Parents receiving the intervention were later interviewed for their stress, child emotions, and feedback about the service using structured surveys administered by psychology graduates. The trainees delivering the intervention were requested to provide their feedback as a written qualitative open-ended narrative. These narratives were analyzed using an inductive approach.

**Results:**

The survey was conducted with 223 families with about half of the sample having children under 2 years of age. Forty-five percent of parents reported play intervention to be one of 3 key factors in improving their experience during hospital stay. Only 5% of parents reported feeling stressed about the child illness after the intervention. Ninety to 96% parents felt respected, listened to, and understood by the therapists. Thematic analysis of the feedback by trainees indicated the internship to be a useful experience and a new avenue for professional life whereas physicians appreciated the interventions.

**Conclusions:**

The authors conclude that psychology trainees can feasibly deliver a play-based intervention under supervision for reduced stress in children and their parents during hospitalization with mutual benefits.

**Supplementary Information:**

The online version contains supplementary material available at 10.1186/s40814-021-00781-8.

## Key messages regarding feasibility

- What uncertainties existed regarding the feasibility?

Since the intervention was being implemented in a tertiary care setting in a LMIC, there were specific uncertainties. Randomization of groups into intervention and control arm was not done due to ethical and logistic concerns about withholding a service from admitted children/caregivers when realizing the intervention was a need. Other uncertainties included (1) the level of training/supervision that would be required for utilizing psychology undergraduate level trainees to deliver the intervention, (2) funding for resources and support from the university hospital and service-line chief for the quality improvement service, and (3) pediatric physicians’ buy-in and confidence in the program.

- What are the key feasibility findings?

It is feasible to utilize psychology trainee students and interns to deliver the play-based psychosocial intervention for hospitalized children and their parents in a private, tertiary care setting. The program allows the trainees an opportunity to learn clinical skills cutomized for hopsitalized children. The activities and protocols are simple, engaging, and perceived to be effective in reducing parental stress and child distress. Most parents felt valued, respected, and cared for during their child’s hospitalization as a result of the support offered to them through the intervention. Paediatric physicians also appreciated the psychosocial outcomes of their patients.

- What are the implications of the feasibility findings for the design of the main study?

Hospital leadership buy-in and support from the outset is imperative for engagement of physicians who may not be keen to uptake psychosocial interventions in the LMIC. Hospitals are complex settings, and one of the most valid methods of testing care-based interventions for in-house facilities. Broader frameworks of developmental care are required that are tailored to suit the needs of each individual family receiving services from the health care system. Further investment for following up with families post-discharge and for connecting children with additional needs to specialized outpatient therapeutic support is needed.

## Background

Data from hospital admissions in the USA in 2012 indicated 2 million hospital stays for children aged 17 years and younger [1] while a study from the UK estimated that pediatric admissions represent 9.9% of the child population aged under 5 years each year nearly [2]. Hospitalization is a stressful time for children and their families with disruption in their routines and uncertainty of their condition, separation from family and community, and painful procedures (up to 6 procedures on average per child) and loss of control [3]. Young children with limited understanding of their condition and the procedures find it difficult to cope and if not intervened experience of pain during hospitalization can have life-long impact on their psychological well-being with children displaying negative behavior like apathy, withdrawal, and sleep disturbances [3]. This is especially concerning for infants as such painful experience can threaten to alter pain pathways and may experience heightened pain sensation throughout life [4].

Recognizing the need of promoting emotional resilience and emotional safety, high-income countries stipulate that all children should have play and stimulation while in hospital, e.g., in the UK, the National Association of Health Play Specialists (https://www.nahps.org.uk/) recommend play while in the USA, American Academy of Pediatrics, recommend use of services by Certified Child Life Professionals (CCLPs) [5] for reduced stress in children and greater experience of families [6]. These professionals undergo rigorous training embedded in developmental psychology, family systems, and therapeutic medical play to create an enabling environment for children and their families during their stay for hospitalization [6]. A recent review of CCLP interventions with young children indicated play-based techniques helped them cope, improved engagement, willingness to eat, and they reported feeling of mastery and growth while parents reported greater satisfaction with services and reduced distress over time. Additional financial benefits were found due to reduction in procedures and overall length of stay and hence lower costs [4].

As evident from literature, specialized play-based intervention not only benefits children and their parents, but also has the potential to affect the healthcare industry by improving the quality of care [7, 8]. This is a dire need in low- and middle-income countries (LMIC) where quality more than access is the largest cause of morbidity and mortality [9]. Despite the recognition of urgent need, unfortunately, no dedicated service-oriented program exists in LMIC for hospitalized children and families owing to many reasons including shortage of resources, physician-centric model, and lack of specialized training for such personnel. Addressing the question will require not just effective but also feasible, scalable, and sustainable intervention models maximizing advantage of existing services and programs with similar scope.

In LMIC, there is considerable focus on early childhood development (ECD) to promote development of disadvantaged children with a critical focus on the early years via health sector. The main risk factors in these setting include poverty, malnutrition, and lack of stimulation which hinder the children to meet their developmental potential. Similar to CCLP requirements, ECD interventions employ play-based framework to nurture parent-child interactions derived from principles of developmental psychology, family systems, and public health [10]. The Nurturing Care Framework (NCF) [11] calls for a stable environment for children for learning and development through responsive care and emotional support (UN, n.d.). Nurturing care has inter-related components than can be applied to hospital setting, including: caregiving (health and psychosocial needs), responsiveness (parental sensitivity and sensitivity and responsiveness when child is irritable or feeling low), nutrition (improve appetite of sick children and encouragement to take appropriate food), stimulation (singing, talking, playing, laughing), and safety (the need to feel safe and secure in a new environment). NCF calls for enabling families to providing nurturing care to their children through strengthening their relationships with the children. It also emphasizes empowerment of vulnerable families who may need additional support [12]. Engaging with families to meet their needs also requires a skilled workforce. A brief summary of the framework has been added as a supplementary file.

The field of ECD provided an excellent opportunity of leveraging the similarity of theoretical framework to address the specific needs of hospitalized children and their families in a tertiary care hospital in a Pakistan. The authors sought to explore the potential of utilizing clinical psychology students during their apprenticeship as specialist workforce to improve care for hospitalized children when delivering a play-based intervention. These students were selected to take advantage of their knowledge and competency-based training pertaining to human interactions, therapeutic relationships, and play therapy. The objective of the study was to test the feasibility and acceptance of the intervention employing play to strengthen parent-child interactions as a means of reduced stress during hospitalization. The study aimed to address the following research question: is a play-based psychosocial intervention delivered by psychology trainees for reducing stress in young hospitalized children (birth to 6 years) and their families in a tertiary care health setting in Pakistan feasible and acceptable to key stakeholders?

## Methods

### Site and sample

This quality improvement (QI) initiative was conducted as a feasibility study in the pediatric ward of Aga Khan University Hospital (AKUH), Karachi, Pakistan—a 700-bedded tertiary care private tertiary care and teaching hospital located in the largest and most populated (~ 28 million people) city of Pakistan serving two provinces. Academic clinical programs form an important part of the service. The pediatric ward has approximately 120 beds with about 8000 yearly admissions and acute illnesses; mainly respiratory infections constitute for 70% of the admissions in the general ward with the average length of stay of 3–4 days. The nurse to bed ratio is 1:6; they are not trained to use play as a distraction with sick children nor is any cadre available to do so. Any child and the family up to 6 years of age admitted in the general ward with acute illness were eligible for the intervention. Approval was sought from the Ethical Research Committee of the Aga Khan University (AKU) prior to commencing the study.

### Intervention

#### Development

A survey was conducted prior to the intervention with 221 caregivers of hospitalized children to evaluate factors affecting parental stress during hospitalization, child emotions and behaviors, and parental knowledge and practices of ECD (Table 1). Parental Stress Survey form related to child illness was informed by previous work by Tehrani et al. [13] and Yang et al. [14]. Responses were categorized and rated according to a Likert scale of five degrees: extremely stressful, moderately stressful, mildly stressful, not stressful, and not applicable. An open-ended item was also included requesting to share their experience and what they appreciated the most during their stay. Child behaviors and emotions were measured using items from the Parent Stress Questionnaire used by Yang et al. [14], a parent-reported measure of child behaviors across 10 different items. Items were rated as experienced most of the time, some of the time, not at all, or not observed. Demographic data was also collected using a structured interview, covering education of the caregiver, language spoken at home, and area of residence and knowledge about child development. The Caregiver Knowledge of Child Development Inventory (CKCDI) was used to gauge maternal knowledge and practices about child’s development [15]. An examination of the CKCDI has revealed the internal consistency of 0.61 and higher maternal education to be a predictor of a higher score indicating validity [15]. The results indicated factors related to the child illness especially seeing the child in pain (79.1%) and the child’s irritability (59.1%) were perceived to be the most stressful. Out of a total score of 20 on the maternal knowledge survey, the mean score at baseline was 6.46 ± 0.44. With respect to practices, the data indicated that the most popular activity was playtime (60% of mothers and 74% of fathers engaged in this), followed by singing songs (58% of mothers, 33% of fathers) and reading books (27% of mothers, 40% of fathers). This highlighted the need for educating parents about play activities.

**Table 1:** Socio-demographic characteristics of pre-intervention participants

	***N*** = 221
**Age (months)**	
0–12 months	176 (79.6)
13–24 months	32 (14.5)
25–36 months	13 (5.9)
37–60 months	0 (0)
**Gender (*N*, %)**	
Male	130 (58.8)
Female	91 (41.2)
**Disease group**	
Acute illnesses	85 (38.5)
Cardiology	28 (12.7)
Neurology	8 (3.6)
Neonatology	46 (20.8)
Surgery	11 (4.98
Neonatology	28 (12.7)
Other	15 (6.8)
**Length of stay (days, median)**	4
**Maternal education**	
Primary	20 (9.1)
School	49 (22.2)
College	38 (17.2)
University or above	107 (48.4)
Missing	7 (3.2)
**Maternal knowledge (mean±SD)**	6.5 ± 0.4
**Language (*N*, %)**	
Urdu	116 (52.5)
Sindhi	44 (19.9)
Other	61 (27.6)
**Residence**	
Within the city	154 (69.7)
Outside the city	67 (30.3)

Data presented as mean ± SD, median or N(%) as applicable

The survey led the investigators to conceptualize a play-based parenting intervention for reduced stress in young children as parents were not well-equipped to use play material for young children’s learning given the role of play for reducing anxiety and trauma in children thereby also reducing caregiver s’ anxiety [16]. Furthermore, play-based care allows healthcare staff to gauge a child’s developmental milestones status and screen for any additional requirements of the child and their caregivers [17].

**Fig. 1:**
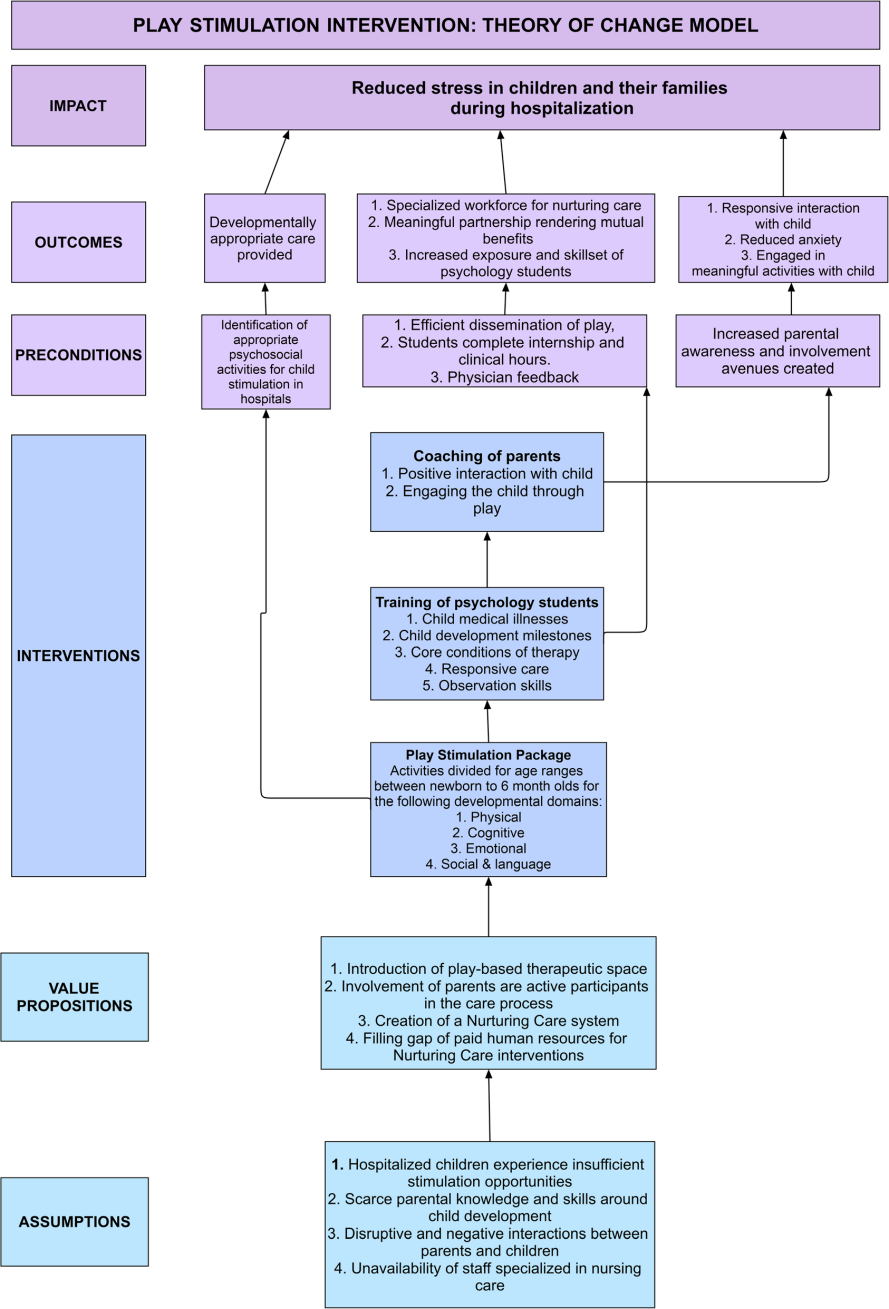
Theory of change model of the intervention

The survey was followed by a theory of change (ToC) model was developed for the intervention clearly outlining family-centered care service whereby parents of children birth to 6 years old were to be coached by psychology trainees for nurturing interactions with their children with no charge to the family (Fig. 1).

The intervention was informed by principles of Nurturing Care Framework (NCF) [10, 11] and play therapy [18]. The scope of NCF was expanded and customized the content, training, and delivery approach to cater to the specialized health and medical needs of the beneficiaries. The intervention approach was borrowed from the *Care for Child Development* module developed by the UNICEF/WHO [19]. The package provides guidance to healthcare providers to help parents to enhance responsive interactions with young using play. The intervention is aimed at strengthening the relationship between parent and child while also providing stimulation. For the intervention package, activities were designed after an extensive literature review of play that helped children in regulating and reducing their stress via play-based nurturing interactions [2]. A manual was created covering four domain of development: physical, cognitive, emotional, and psychosocial and communication across five different age groups: (1) newborn to 6 months, (2) 7–12 months, (3) 13–24 months , (4) 25–36 months, and (5) 48–72 months. The emotional domain was kept separate and had a significant number of activities. Since hospitalization is an emotionally taxing event for both children and families the investigators felt it needed attention on its own. The activities from this domain were informed by the principles of play therapy which uses play as a medium of expression for children. Play is also helpful in assessing emotional distress while building therapeutic relationship with the child. The intervention materials are summarized in Table 2.

**Table 2:** Intervention materials

Resources	Materials
Intervention job aides	1. Intervention manual 2. Milestone checklist 3. Play activities checklist 4. Storybooks log 5. Cartoon videos log 6. Toys
Standard operating procedures	1. Guidelines for individual sessions 2. Guidelines for group sessions 3. General standard operating protocol 4. Infection control protocol
Training package	1. Training manual for trainees 2. Training manual for nurses
Monitoring and evaluation forms	1. Session information: activities conducted, and psychosocial profile 2. Supervisor observation checklist 3. Case study template 4. Intern evaluation template 5. Physician report template

#### Recruitment, training, and supervision of trainees

A memorandum of understanding was made with a psychology department of a humanities university for formal approval of internship of their students following meeting of the PI, the service line chief with the Director of the Psychology Institute at the university. The trainees were recruited through interviews by the clinical supervisors (study investigators) and were evaluated for their interest in working with children along with other core skills such as communication, rapport-building, family counseling, and motivational interviewing. A batch of students was required to rotate for 6 weeks and complete 100 h before the next batch joined. The internship hours contributed toward their apprenticeship requirements.

The trainees received a 4-day training in their first week. The first 2 days were focused on imparting the theoretical concepts of the core concepts of therapy, importance of play, behavioral modification techniques, discrete observations of children and parents, familiarization with the intervention materials, and forms and protocol for disinfection of toys. This was followed by practical demonstration and practice. Trainees observed their supervisor conducting the session after which they were asked to take sessions in pairs first and then independently. Once trainees started working independently, they had weekly in-depth supervision meetings for 2 h end of the week (Friday). The meeting entailed discussing difficult cases, countertransference, and grievances to keep building their knowledge and skills. Once a week, interns were also observed via supervision observation checklist developed for the study. The checklist was focused on different skills, e.g., intervening with an individual child required being prepared for the session, rapport building skills, structuring the session, communicating the purpose of the session and recommendations to the child and the family. Other skills included application of core conditions of therapy, setting boundary, and being sensitive to cultural difference with the families. The feedback was shared with the trainees in the in-depth debriefing meeting. At the completion of the internship trainees were expected to submit a case study to gauge their clinical knowledge. The case report was similar to the assignments in clinical psychology whereby detailed case history of a selected client is reported along with interventions completed and recommendations. The case report was evaluated by the clinical supervisor and also shared with their university. An evaluation report was also submitted to their university supervisor highlighting strengths and areas of improvement of the trainees. The evaluation reported areas of their technical domains: conceptualization, assessment, and intervention skills and personal and professional domain: communication and interpersonal skills, being a team player and respect for cultural diversity, informed by the framework for internship by American Psychological Association [20].

#### Intervention procedures

While originally designed to be a combination of group and individual sessions, groups were not feasible within the setting owing to space constraints, infection control guidelines, children’s conditions, and other activities of priority to the child’s health, e.g., medication timings or lab tests. The intervention sessions were delivered at bedside, 4 days a week as per the trainees’ university schedule. A standard protocol was developed for the intervention for selection of activities and coaching the parents to play with their children using these activities intending to strengthen their relationship. Families were approached for the play-based sessions if the child was awake and the physician round had been completed so the family would be at ease. Each session lasted between 20 and 40 min depending on the child’s developmental stage and mental state. The session was designed to include an ice-breaker activity followed by main activity and closure selected based on the developmental needs of the child assessed on observation. The parents were advised to carry out the activities with the child. Between the sessions, a thorough activity of disinfecting toys was ensured, based on AKU DIPHE (Department of Infection Prevention and Hospital Epidemiology) recommendations. At the end of the day, the supervisor would complete rounds with the trainees and answer any queries that the parents may have. The round was also an opportunity to assess parental satisfaction with the intervention and note any gaps in the recommendations provided by the trainee. Notes were added to the medical record file of the child for review by the physician by the supervisor. Subsequently, the supervisor also shared a detailed report of the child’s developmental profile with the referring physician. The report was an assessment of the child based on clinical observations including mental state examination of the child (cognitive, mental and functional status) and developmental status. Intervention activities conducted, response of the child, and recommendations were also documented. A weekly monitoring form was created toward the end of the study to understand the on-going trends and intervene if needed.

### Feedback from stakeholders

Several methods were used to seek feedback from the participants: parents, trainees, and physicians.

#### Parents

Data was collected as a survey by two trained research assistants with background in Psychology between October and December 2019. The inclusion criteria were (1) children’s age (1 month to 6 years), (2) hospital stay of at least 24 h, (3) children admitted in general ward, and (4) having received at least one play therapy session. All eligible families were approached for feedback after reviewing the list of children admitted in the ward who met the criteria. The exclusion criteria included all children above 6 years. The families were approached by the data collectors for feedback in the ward. The primary variables of interest included parental report of reduced negative emotional affect of the child and stress in parents using the same measure as in the pre-intervention survey. Parental feedback about the intervention was also sought on a structured form on a sub-sample prior to discharge by the trainees.

#### Trainees

Trainees played a central role in the program implementation that was premised on partnership with the education sector, and hence, their feedback was solicited to gauge benefits they accumulated. They were required to provide an open-ended qualitative narrative feedback about strengths and areas to improve once their internship ended.

#### Physicians

Recognizing that physician role buy-in was important for feasibility of the intervention their feedback was also sought. Consultant physicians were requested to provide feedback via email when a report of the child seen was shared with them. Some of them also shared their experience on the departmental social media group.

### Data analysis

Data entry operators compiled the collected data on Microsoft Office Excel. Data was entered and analyzed using Statistical Package for Social Sciences (SPSS, version 15.0) by an independent statistician. Demographic variables included child gender, child disease group, length of stay, maternal education, maternal knowledge of ECD, residence, and language spoken at home. Values were presented in the form of mean ± SD and frequencies with percentages for continuous and categorical variables respectively.

Thematic analysis was conducted for qualitative data of trainee narratives and physician feedback using an inductive approach. The narratives were coded by two team members (first two authors) closely involved with the implementation of study. The scripts were coded by two individuals independently and then finalized after agreement in a face-to-face meeting. Codes were also mutually agreed in the agreement meetings. For quality assurance, a third member, an experienced developmental psychologist and independent of the implementation reviewed the codes. Her understanding of the underlying developmental processes provided a different lens to the analysis. Moreover, the first author has considerable experience with qualitative methodology to evaluate implementation of parenting interventions allowing to pick up nuisances. A thematic map was created to further evaluate the relationship between codes.

## Results

### Parental feedback

Data of 223 participants who received intervention was analyzed for the study (Table 3). About a third of children were in the age group of birth to 1 year (31.8%), were slightly higher in number of females (56.95), and majority were from the acute illness group (72.2%). The median length of stay was 4 days. Out of a total score of 20 on the maternal knowledge survey, the mean score was quite low (4.3 ± 0.29).

**Table 3:** Socio-demographic characteristics of participants exposed to intervention (*N* = 223)

Characteristic	***N*** (%)
**Age (months)**	
0–12 months	71 (31.8)
13–24 months	60 (26.9)
25–36 months	34 (15.2)
37–60 months	58 (26.01)
**Gender (*N*, %)**	
Male	127 (56.9)
Female	96 (43.05)
**Disease group**	
Acute illnesses	161 (72.2)
Cardiology	13 (5.8)
Neurology	15 (6.7)
NICU	2 (0.9)
Surgery	30 (13.5)
Neonatology	0 (0)
Other	2 (0.9)
**Length of stay (days, median)**	3
**Maternal education**	
Primary	39 (17.5)
School	25 (11.2)
College	37 (16.6)
University or above	114 (51.1)
Missing	8 (3.59)
**Maternal knowledge (KAP, mean±SD)**	4.3 ± 0.29
**Language (*N*, %)**	
Urdu	128 (57.4)
Sindhi	32 (14.4)
Other	63 (28.3)
**Residence**	
Within the city	154 (69.1)
Outside the city	69 (30.9)

Table 4 shows frequencies and percentages of parental stress due to child illness. Results indicate items marked as “extremely stressful” by parents and “most of the time” for children. Most negative emotions in parents stemmed from seeing their child in pain or distress (6–7%), as well as uncertainty about the child’s health in the future (10.6%) and the possibility of a relapse (14.5% marked as “extremely stressful”).

**Table 4:** Parental stress related to child illness and child expression of negative emotions (N=223)

	***N*** (%)
**Stress perceived by parents**	
The child appearance, e.g., lethargic, weak or pale	6 (2.6)
Prolongation of hospitalization	9 (3.8)
The severity of disease	6 (2.6)
Child’s inability to eat	24 (10.2)
Uncertainty about future of the child’s medical condition	25 (10.6)
Fear of relapse	34 (14.5)
Child irritability and crying	17 (7.2)
Child’s pain	14 (6)
**Emotion/behavior experienced by the child**	
Rebellious	50 (21.3)
Crying	16 (6.8)
Demanding	42 (17.9)
Frightened	32 (13.6)
Anger	27 (11.5)
Restlessness	58 (24.7)
In pain	47 (20)
Sad	94 (40)
Confused	82 (34.9)
Inability to talk/cry	49 (20.9)

Results are presented as *N* (%) for the “extremely stressful” category for parents and “most of the time” for children

About 45% of the parents (*N* = 94) reported play intervention as one of the three key factors that made their experience positive during the stay. Some parents reported that play helped to distract their child from the pain, helped the child calm down, while some suggested that play should be an integral part of pediatric care services. One parent (interview ID 44) reported “The play [activity] is effective. It was the only thing that made me feel good about my experience [in the hospital].”

Parent feedback collected by the trainees also showed similar trends. Though only 20% of the parents were captured due to time constraints at discharge, majority reported being satisfied with the intervention. Ninety-two percent agreed/strongly agreed that they felt satisfied, that they were listened to and respected by the therapist, and that the therapist cared about their child’s well-being; 96% agreed to feeling comfortable talking to the therapist and felt relaxed after their session; 94% felt that their relationship with their child was strengthened; and 85.5% agreed/strongly agreed that they were able to focus on their children as well thanks to the program (not applicable to 10% of participants with an only child).

### Trainees feedback

Though designed initially only for local students pursuing a Master’s degree, the program also attracted several students (of Pakistani origin) from international universities and other undergraduate students with social sciences background. Out of 33 trainees, 9 were undergraduate level students and 24 were enrolled for their Master’s degree; 5 were international students. One of the trainees was male, and 32 trainees were female.

**Fig. 2:**
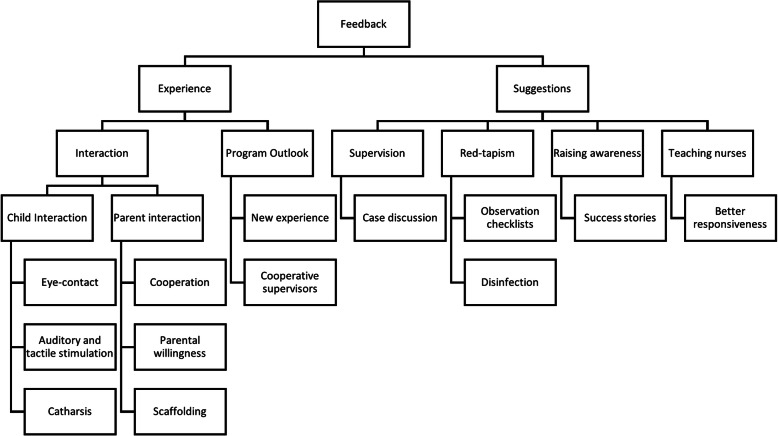
Thematic analysis map of trainee feedback

Findings from analysis of trainees’ feedback (summarized in Fig. 2) indicated that they appreciated identifying new avenues in clinical psychology and realizing the importance of parental interaction and the importance of their cooperation and willingness.

A trainee (October 2019) stated, “The work experience and exposure was remarkable; the supervision provided was incredibly supportive and helpful. Mistakes were highlighted, and weekly feedback was so beneficial. Initially, it was a bit challenging but over time, it became fun to work with different types of audience. Every client helped us to grow in their own way.”

They also reported that this was a “new, much-needed exposure” and that holistic care should be a part of every hospital in Pakistan (“The internship made me realize the need for child therapists and psychologists in the Pakistani medical system to provide holistic treatment that caters to the needs of both, the parents and the children”, trainee, July 2018). One of the trainees’ sister was admitted to the ward for 10 days during the internship. The trainee practiced play therapy techniques with her sister and reported that she could “vouch for the techniques…they enhanced my skills as a person and as a psychologist. The activities played a significant role in instilling hope in my sister, as well as improving the bond between us.”

Some of the success stories included feedback from the international students. They felt the principles of play-based interventions resonated with their work in the USA and Canada—“I am now back in the USA. I will be working as a volunteer for play therapy at the Boston Children’s Hospital. I am so impressed that this concept has been introduced at AKUH because I see that play and creative arts therapy is an important part of healing. As [an AKUH pediatrician] said, once the play therapists and psychologists go and talk to the patients, that’s when the medicine starts working.” (Trainee, September 2018).

The other international student trainee (May 2019) remarked, “Being born and raised in East Africa, and now living in Canada, even with a wide range of experiences in certain areas, I would have never found an opportunity such as this one anywhere else … [it] has opened my eyes to the path I want to take in the future … Being here and undergoing all the responsibilities has assured me that I truly enjoy helping others, and teaching/training medical practitioners in order to increase their depth of knowledge in a holistic manner. This way there is a larger population of individuals who are well versed in multiple areas including play therapy and can help more patients daily, compared to one or two of us who are currently working alone. Overall, it has definitely been an eye-opening and life-changing experience for which I will be eternally grateful.”

Suggestions for improving the program included reducing the time duration of disinfection (“[better to] assign nurses or any other [lower level] staff who could be responsible for disinfecting the toys properly… thereby giving interns the time and opportunity to choose an activity or toy in accordance with the needs of the next patient”, trainee feedback, July 2018) and including a variety of toys to cater to the patient population from different parts of the country (“…increase the kinds of toys and interventions available, especially for kids older than 10 years. Adding picture books or simple story books, with languages like Sindhi, might also be beneficial” trainee feedback, December 2019).

Raising awareness was also emphasized upon and the role of all health providers for nurturing interaction with children. The students felt the success of the program could be disseminated widely given potential benefits. A trainee (July 2018) suggested, “parents should be more involved in the activities, psychoeducation of parents should be the main focus. Staff (primarily nurses) should be trained in basic therapeutic techniques, helping them to create a positive bond with the patients, and supporting children when the therapist is not available. This will reduce patient and parent anxiety in the hospital.”

### Physician feedback

Core themes of gratitude, patient support, improvement, detailed feedback, and recommendations were identified from physician feedback. Most of the physicians who responded on email were thankful for the observation reports that were sent to them and were grateful for the continuous support. They also observed changes in children, such as decrease in anxiety and increase in curiosity and social interaction. Physicians also recommended that children and families would benefit from stimulation due to chronic conditions. Overall, the implementation of the program was appreciated by the consultants as well.

A pediatric oncologist appreciated that “It’s very encouraging to have this kind of service available in The Children’s Hospital,” while a gastroenterologist noted that the “explanations about the child’s psychosocial profile are really helpful.” Some physicians requested a long-term planning “Maybe we can sit down and develop a plan where we have someone who works very closely with our patients as these patients require years of therapy in certain situations.”

A cardiologist noted on the departmental Facebook group (April 2018): “… yesterday I gave a consult to [the psychologist] for 2 patients. One is a post-op cardiac patient who yesterday morning was quiet and not interactive. The parents were extremely worried about her change of personality. Today after just one session by [the psychologist] I could not recognize the child – interactive, playful, fearless. She kept pointing to this note by [the psychologist] (a teaching note for the parents) and telling her dad to follow the instructions! Thank you so much for starting the play therapy service in the Children’s Hospital.”

An oncologist (May 2018) responded to the above comment: “Agree 100% with this … seeing [the psychologists] interacting with our patient was heartening… play therapy project is much needed by the Children’s Hospital – seeing Child Life work so closely with oncology patients, I always felt this void here until now. Appreciate [the investigators] for creating this.”

### Key feasibility findings

Recommendations for modification to the program and the way forward are summarized in Table 5.

**Table 5:** Key findings from feedback of parents, trainees, physicians

Domain	Key findings/way forward
Randomization	Randomization can be done; one possibility is to examine play activities, with and without parental coaching stratified by disease groups as either acute or complex. Another possibility is to ranomized groups for follow-up support post-discharge. A group with no intervention will not be possible as all children/families will be offered this service upon admission into the ward and withholding will be unethical.
Intervention implementation	Dedicated senior staff are required to oversee the execution of the program and follow a formalized process of monitoring and evaluation as planned for quarterly meetings. Though started as a pilot service, the program evolved very quickly and staff were stretched thin to incorporate regular feedback and changes.
Intervention content	No change is recommended for the content. One addition can be to encourage special education services for children with prolonged stay or repeated admissions.
Intervention mode	Individual bedside sessions are feasible, particularly compared to groups, due to space constraints and privacy concerns. Groups also enhance risk of infection.
Intervention delivery staff	Students, preferably enroled in Master’s programmes are suitable for the intervention delivery. Undergraduates can be utilized but not as core delivery staff.
Intervention delivery forms	The content needs simplification and length of forms needs to be reduced. An additional form is required for quick screening of developmental and emotional issues to help trainees frame their intervention plan. The forms need to be co-designed with the key stakeholder who will use the data, e.g. physicians for child health outcomes and university supervisors for academic outcomes of the trainees.
Engaging trainee students	Continue to share reports with university management. Make more efforts to engage university supervisors through in-person meetings. It is an important aspect to assure quality and professionalism by students while imparting a sense of accountability. Ideally, after every rotation so lessons learnt from one should be incorporated into the next rotation.
Engaging physicians	Individual reports are very time-consuming and feedback on emails may not always be received. Having in-person monthly meetings while sharing trends with physicians aggregated by disease groups can be more effective. Incorporate their feedback into the on-going programme.
Continuation of services for children	Children with additional needs should be identified during the intervention and connected for out-patient follow-up.
Evaluation	Resources are needed for meeting families post-discharge and collecting their insights about the intervention. Meeting minutes with university supervisors and physicians can also add to the process evaluation of the intervention. A log of supervisor observations can be maintained covering their weekly in-depth meetings and rounds. To determine effectiveness, a trial is recommended.

## Discussion

The findings indicated that a play-based psychosocial intervention following principles of Nurturing Care Framework and play therapy in a hospital setting and utilizing trainee students was acceptable as parents perceived it to reduce children’s affective state and reduce their own stress related to child illness. The finding aligns with literature from high-income countries where play-based techniques have consistently demonstrated effects for reduced parental anxiety and more positive affects in hospitalized children [21]. Coaching parents could have contributed toward child’s experience of positive feelings as provision of warm and responsive care [22–24]. Family-focused interventions lead to satisfaction with healthcare services as well as better coping in children and parents [25]. It has also been found out that anxiety in children also decreases during hospital play with and without family members being present [26]. However, teasing out these differences was not possible with the current study design but could be considered to design a trial in the future. With regard to acceptance, about half of the parents rated it as one of the three factors that added value to their experience during hospitalization. This is encouraging and a low-cost investment could improve the patient experience while adding to the reputation of the service providers. Moreover, from the hospital’s perspective, an important outcome of the intervention has been inclusion of parent-child interaction as part of nursing assessment of the child at admission and adoption of the play-based intervention package as a default service by the hospital. This will help children with becoming comfortable earlier on and getting familiarized with medical procedures while helping to understand the effects of hospitalization and disease on children [27].

An important observation in the study was the duration of sessions ranging from 20 to 40 min. We deduced from our experience of implementing the intervention that children who were sick took relatively longer to engage in the activity than children who are not sick (e.g., in the community). The response of the child was dependent on his/her affect and also parental engagement. The aim of the session was to enhance parent-child interaction and continue the session till both found it mutually enjoyable interaction. This may have implications for scalability but we feel it is important to let parents and children have enough time to engage in meaningful interaction for the intervention to be effective.

The study yields possible pathways for the continuation of positive ECD practices in a hospital setting with a risk of compromised child development especially in the absence of specialized training programs by leveraging existing resources. Using students as a workforce opens new avenues for them to complement healthcare. Exposure to early childhood training, such as teaching training, internship influences career choice and perspective on interaction with children [28]. Youth, especially females, can be important agents for community mobilization, social awareness, and social transformation [29].

Moreover, such an approach can also support attainment of several SDGs. SDG 3, supporting holistic care as an essential quality healthcare service, is achieved when hospitalization may push behind these children academically, but play-based psychosocial intervention may help them acquire new skills and get equitable learning opportunities promising inclusivity and delivery of SDG 4, i.e., quality education. This also encapsulates SDG 4—development of youth skills for career development. The female-led model requiring compassion as a core ingredient also supports gender equity (SDG 5, i.e., gender equality) where communities see it as a women’s job and is accepted by families. SDG 17 calls for establishing partnerships for strengthening the means of implementation and ensuring sustainable service development. Partnership with the education sector opened opportunities for the investigators to utilize the skills of university students, specializing in psychology. The investigators believe that universities can play a major role to fill the gap of paid human resources for psychosocial interventions.

A potential limitation was no direct observational assessment of child behaviors, eliminating any possible biases of the parent report. Another limitation was that in-depth interviews were not feasible with the stakeholders due to resource and setting constraints. Qualitative interviews would have yielded valuable insights. However, investigators were integrally involved with the study and participants and documented their reflections and feedback. An interesting perspective would be to follow-up children later to explore if the parenting skills imparted during the hospitalization were continued at home.

While promising, the results cannot be generalized to a different setting. This was a private care hospital where overall adequate care was being provided compared to other public care settings in the country which can also impact parent stress during child’s hospitalization. However, the authors are also working to replicate the model in less developed parts of the country. Moving forward, courses can be designed in collaboration with training centers for early childhood education to create a specialized workforce for healthcare settings. Further analyses would require understanding parent-child interaction as a disease modifier to advocate for integration of such interventions in LMICs health sector. Nonetheless, the study helps in understanding the role that NCF plays in a hospital setting. Through this pilot, we demonstrated feasibility of delivery of play-based interventions by trainees in improving patient experience in an acute setting. This was done through partnering with a local university. Such an approach can be cost-effective in resource-constrained health systems, allowing the integration of psychosocial aspects of well-being and development into health care.

## Conclusions

Litarature from high-income countries suggests that provision of play-based services in hospital setting reduces anxiety and stress in both parents and children given caregivers of hospitalized children are also in need of emotional support. These psychosocial needs are often met by different professionals in a hospital setting in high-income countries. In LMICs, ECD practitioners and psychologists can use play-based techniques for the provision of psychosocial support to children and their families in a hospital in resource-constrained settings. Direct involvement of parents in providing psychosocial stimulation children decreases stress in both in a hospital setting, which is congruent with the findings of ECD research. Trainee students can deliver psychosocial support interventions under supervision in these settings and simultaneously gain clinical experience from the programme. Utilizing trainees for play-based psychosocial support for hospitalized children is feasible; however, to determine intervention effectiveness, further investment of resources and a randomized trial is required.

## Supplementary Information

**Additional file 1.** Intervention Framework

## Data Availability

Data and intervention materials (manuals and forms) can be made available upon request to the corresponding author.

## Abbreviations

AKUH: Aga Khan University Hospital, Karachi
APA: American Psychological Association
CCLP: Certified Child Life Professionals
CKCDI: Caregiver Knowledge of Child Development Inventory
DIPHE: Department of Infection Prevention and Hospital Epidemiology
ECD: Early childhood development
LMIC: Low- to middle-income countries
M & E: Monitoring and evaluation
NCF: Nurturing Care Framework
SDG: Sustainable Development Goals
SOP: Standard operating procedures
ToC: Theory of change model
