# Supplementary material for: Development and feasibility testing of a play-based psychosocial intervention for reduced patient stress in a pediatric care setting: experiences from Pakistan
Source: Pilot Feasibility Stud. 2021 Mar 6;7:63. doi: 10.1186/s40814-021-00781-8 (PMC7936486; doi:10.1186/s40814-021-00781-8)
Supplement: Supplementary file 1 — Additional file 1. Intervention Framework [file 40814_2021_781_MOESM1_ESM.docx]

**Supplementary File
Intervention framework**

*Box 1: Development of the intervention model*

| ***ECD Practices as Theoretical Influence***  The thesis on which behind Early Childhood Development (ECD) practices rest informed the development of play-based intervention model in a hospital setting. Similar to Child Care Licensing Program (CCLP) requirements, ECD interventions employ play-based nurturing interactions derived from developmental psychology principles, family systems, and public health^1^.  ***Literature Review Findings***  To develop an evidence-based package, a literature review was conducted to explore potential psychosocial interventions effective in reducing the negative emotions and behaviour. The findings revealed the following main problems experienced during hospitalization^1^:   - lack of identity - difficulty in communication, and - parental stress that impacts children’s stress   Studies showed that techniques like socio-dramatic play, symbolic play, and playing with objects helped children in regulating and reducing their stress via play-based nurturing interactions^2^  The final package was developed for young children between birth to 6 years, borrowing principles from Nurturing Care Framework and Play Therapy.  ***Nurturing Care Framework***  The nurturing care framework (NCF)^3^ calls for a stable environment for children for learning and development through responsive care and emotional support (UN, n.d.). Nurturing care has inter-related components than can be applied to hospital setting, including:   - caregiving (e.g. health and psychosocial needs) - responsiveness (e.g. parental sensitivity and sensitivity and responsiveness from nurses) - nutrition (improve appetite of sick children) - stimulation (e.g. singing, talking, playing, laughing, and - safety (the need to feel safe and secure in a new environment)   The research team expanded the scope of NCF and customized the content, training, and delivery approach catering to beneficiaries’ specialized health and medical needs.  ***Play Therapy***  Play therapy uses play as a medium of expression for children and helpful in assessing emotional distress while building therapeutic relationship with the child. A recent review of play-based interventions with young children indicated following benefits^4^:   - coping - improved engagement - willingness to eat - feeling of mastery and growth - greater parental satisfaction with services, and - reduced parental distress over time   Additional financial benefits were found due to reduction in procedures and overall length of stay and hence lowers costs. |
| --- |

**References**

1. Nahar B, Hamadani JD, Ahmed T, et al. Effects of psychosocial stimulation on growth and development of severely malnourished children in a nutrition unit in Bangladesh. *Eur J Clin Nutr.* 2009;63(6):725-731.
2. WHO. Nurturing Care Framework: Why nurturing care? World Health Organization. <https://www.who.int/maternal_child_adolescent/child/nurturing-care-framework-rationale/en/#:~:text=Nurturing%20care%20refers%20to%20a,are%20emotionally%20supportive%20and%20responsive>. Published 2019 Accessed 20 May 2020
3. Britto PR, Lye SJ, Yousafzai A, et al. Nurturing care: promoting early childhood development. *The Lancet.* 2017;389(100064):91-102.
4. Godino-Iáñez MJ, Martos-Cabrera MB, Suleiman-Martos N, Gómez-Urquiza JL, Vargas-Román K, Membrive-Jiménez MJ, Albendín-García L. Play Therapy as an Intervention in Hospitalized Children: A Systematic Review. In Healthcare 2020 Sep (Vol. 8, No. 3, p. 239). Multidisciplinary Digital Publishing Institute.
